# On the Incipient Stage of Cancerous Affections of the Womb

**Published:** 1842-11

**Authors:** W. F. Montgomery


### Selections from American and Foreign Journals.

---

*On the incipient Stage of Cancerous Affections of the Womb.* By Dr. W. F. Montgomery.—In this paper the author directs the attention of practitioners to a stage of cancer uteri which precedes the two usually described by writers.

The *symptoms* are—sharp but comparatively fugitive lancinating pains in the back and loins, across the supra-pubic region, or shooting along the front of the thigh, or sometimes along the course of the sciatic nerve, producing numbness, and not unfrequently debility of the whole limb.

In a large proportion of the cases, there is found a decided fulness, or a distinct tumor in one or other iliac hollow, with fixed pain, and tenderness traceable to, and, as it were, issuing out of the abdominal ring; there is, generally, more or less irritation of the bladder, with dysuria, and the patient often complains of a sensation about the lower part of the rectum, which induces her to think that she is laboring under piles. Menstruation, though in some instances disturbed, is much more frequently quite regular in its returns; but there is apt to be bursts of hemorrhage, either accompanying the discharge, or occurring in the intervals; there is little, or no leucorrhœal or serous discharge, often none; and it is not until the disease has existed for a considerable time, that the appetite is impaired, sleep is disturbed, the flesh becomes softer and wastes, and the countenance pale, and expressive of distress.

On making examination *per vaginam*, the margin of the os uteri is found hard, and often slightly fissured, and projects more than usual, or is natural, into the vagina, and is irregular in its form.

In the situation of the muciparous glands, there are felt several small, hard, and distinctly defined projections, almost like grains of shot, or gravel, under the mucous membrane.

Pressure on these, with the point of the finger, gives pain, and the patient often complains that it makes her stomach feel sick.

The cervix is, in most instances, slightly enlarged, and harder than it ought to be. The circumference of the os uteri, especially between the projecting glandulæ, feels turgid, and to the eye presents a deep crimson color, while the projecting points have sometimes a bluish hue.

There is no thickening, or other alteration of structure in any part of the vagina, at its conjunction with which the cervix uteri moves freely; nor is there any consolidation of the uterus with the neighboring contents of the pelvis; in fact, the morbid organic change appears to be, at first, entirely confined to the os uteri and lower portion of the cervix.

This stage of the affection is, in many instances, *very slow*, lasting sometimes, *for years*, before the second and hopeless stage is established; during this time the patient experiences only comparatively slight and transient attacks of pain, or perhaps only sensations of uneasiness, referred often to the situation of one or other of the ovaries, or about the os uteri, with anomalous tingling along the front and inside of the thighs; these last for a few hours, or a day or two, and then disappear, perhaps for weeks; but again and again return in the same situation, and for a long time are not increased in severity; the patient finds that sexual intercourse now, occasionally, causes her pain, which she ascribes to some deep-seated part being touched, and the act is followed by an appearance of blood; she is, also, often troubled with slight irritability of the bladder; but the appetite, digestion, and sleep, may, for a long time, continue good, and the pulse, generally, gives no indication of the existing disease, or its changes; an observation which will be found applicable to many uterine affections of a grave character; in short, the general health may long remain quite undisturbed, nor has the patient, in many instances, the slightest suspicion that there is any thing seriously wrong with her, nor thinks of seeking for medical aid until she is induced to do so by the solicitations of her husband, or some anxious friend who has become, as she thinks, unreasonably alarmed about her state.

Dr. Montgomery thinks that the first discoverable change in the cases now alluded to "takes place in and around" the muciparous glandulæ, which exist in such numbers in the "cervix and margin of the os uteri;" these become indurated by the deposition of scirrhous matter around them, and by the thickening of their coats, in consequence of which they feel, *at*

*first*, almost like grains of shot or gravel under the mucous membrane.

*Treatment.*—In almost every instance, the treatment should be begun by the *local abstraction of blood*, either by cupping, or by leeches applied to the os uteri, or as near as possible to the organ; and their application will, in most cases, require to be frequently repeated, and should be accompanied by the free use of anodyne fomentations. *Venesection* is not, in general, required. Except there be something specially to forbid its use, *mercury* should be given, in some form, so as to bring the system very gently, but decidedly, under its influence; for which purpose it may be combined with iodine in very minute proportions, with camphor, opium, hyoscyamus, or hemlock; and occasionally by friction, especially where there exists evidence of inflammatory action in the iliac hollow, as already adverted to.

Afterwards, *iodine* or *hydriodate of potash* may be used both internally and externally; and *iron* will be found a most beneficial and powerful agent, especially in the form of the saccharine carbonate, or the carbonate given in the nascent state. The *iodide of iron*, which combines, to a certain degree, the powers of both remedies, may also be used with some advantage in most cases. *Counter irritation* is an agent of very great influence in this complaint, and may be established in a variety of ways, which it is unnecessary to enumerate; but a very effectual mode is by making a small blister over different parts in succession, and keeping it discharging freely for several days, by the application of the French dressing, or Albespeyer's papers.

After the removal of the congestion and organic changes from the os uteri, there remains, occasionally, a sensitiveness of the part, which causes the patient much discomfort, and which will be best relieved by the use of the bath, as above directed, conjoined with anodyne applications to the part, or the nitrate of silver in solution; the best mode of applying which, is by means of a bent glass tube of about an inch in diameter, which the patient can introduce and manage for herself; all that is necessary is, that she would lie on her back, and introduce the tube as far as its curvature, and then pour into the upper end the medicated solution, which will immediately pass to the os uteri, and can be retained there as long as necessary, the tube filling the vagina sufficiently to prevent its flowing away, which is a great advantage.

The patient should be strictly enjoined to avoid every thing that could stimulate the uterus—such as riding on horseback,

&c.; but, especially, she should refrain from indulgence in sexual intercourse. Wine, if used at all, should be of a very mild kind, and very sparingly taken, and the same rule should apply to malt drinks; the stronger kinds of ale and porter should be altogether prohibited.

No circumstance connected with the treatment of this affection requires more scrupulous attention than the regulation of the patient's habits and modes of living; indeed, if this be not very carefully managed, all other measures will most probably be defeated.

In illustration of the foregoing remarks, Dr. Montgomery relates several cases which terminated successfully under the treatment laid down by him. Further researches, however, are required to establish that the nature of the disease is truly cancerous.—*Prov. Med. and Surg. Jour.*, from *Dub. Jour. Med. Sci.*

